# Insight into IL-5 as a Potential Target for the Treatment of Allergic Diseases

**DOI:** 10.3390/biomedicines12071531

**Published:** 2024-07-10

**Authors:** Katarzyna Antosz, Joanna Batko, Marta Błażejewska, Antoni Gawor, Jakub Sleziak, Krzysztof Gomułka

**Affiliations:** 1 Student Research Group of Internal Medicine and Allergology, Faculty of Medicine, Wroclaw Medical University, 50-368 Wroclaw, Poland; katarzyna.antosz@student.umw.edu.pl (K.A.); joanna.batko@student.umw.edu.pl (J.B.); marta.blazejewska@student.umw.edu.pl (M.B.); antoni.gawor@student.umw.edu.pl (A.G.); jakub.sleziak@student.umw.edu.pl (J.S.); 2 Department of Internal Medicine, Pneumology and Allergology, Faculty of Medicine, Wroclaw Medical University, 50-368 Wroclaw, Poland

**Keywords:** interleukin-5, asthma, allergy, biological treatment, benralizumab, mepolizumab, reslizumab

## Abstract

Interleukin-5 functions as a B-cell differentiation factor, but more importantly, in the context of this review, it plays a variety of roles in eosinophil biology, including eosinophil differentiation and maturation in the bone marrow, and facilitates eosinophil migration to tissue sites, usually in the context of an allergic reaction. Given the availability of selective anti-IL-5 drugs such as mepolizumab and reslizumab, as well as the IL-5 receptor antagonist benralizumab, it is worth investigating whether they could be used in some cases of allergic disease. Asthma has a well-documented involvement of IL-5 in its pathophysiology and has clear benefits in the case of anti-IL-5 therapy; therefore, current knowledge is presented to provide a reference point for the study of less-described diseases such as atopic dermatitis, chronic rhinosinusitis, chronic spontaneous urticaria, and its association with both IL-5 and anti-IL-5 treatment options. We then review the current literature on these diseases, explain where appropriate potential reasons why anti-IL-5 treatments are ineffective, and then point out possible future directions for further research.

## 1. Introduction

The term “T-cell replacing factor (TRF)” first emerged in the 1970s [1], referring to the factor inducing the terminal differentiation of B cells into Ig-secreting cells. Over time, it evolved into “B cell growth factor (BCGF)” or “B cell differentiation factor (BCDF)”. However, due to its diverse activities and targets besides B cells, it was eventually termed Interleukin 5 (IL-5) [2,3].

IL-5 has significant effects on allergy and asthma due to its role in eosinophile proliferation and differentiation, which have been well documented [2,4]. To demonstrate the involvement of IL-5 in asthma, anti-IL-5 mAbs inhibited antigen-induced airway hyperresponsiveness and the influx of eosinophils into the airways, as shown in this study [5], among others.

Despite IL-5’s well-recognized role in the pathomechanism of asthma, our aim is to provide a comprehensive overview to establish a baseline for understanding IL-5 involvement in other allergic diseases and the potential therapeutic options for inhibiting IL-5 to stop or at least slow down the diseases. Currently, there are three monoclonal antibodies to choose from: mepolizumab and reslizumab—selective IL-5 inhibitors; and benralizumab—an IL-5 receptor (IL-5R) antagonist. All of the above have proven to be effective and safe, as confirmed by various meta-analyses, clinical trials, and real-life studies [6,7,8,9,10]. To further demonstrate the area of application of these drugs, we have provided detailed data on the structure of these drugs, their dosage, use cases, and effectiveness in other studies.

Moving beyond asthma, atopic dermatitis (AD) is a disease affecting up to 1 in 5 children and 1 in 10 adults [11], characterized by a chronic course of the disease and an increased risk of other allergic and psychiatric disorders [12]. We delve into the potential connection between IL-5 and its role in skin inflammation, i.e., prolongation of eosinophil survival time and induction of eosinophil chemotaxis as a result of increased IL-5 expression [13]. However, the first study conducted in 2005 [14] and subsequent studies [15,16] did not demonstrate the effectiveness of anti-IL-5 treatment in AD. Therefore, we have reviewed and investigated the possible reasons why this is happening and then indicated possible directions of development going into the future.

Subsequently, Chronic Spontaneous Urticaria (CSU), because of its chronic nature and the lack of a specific triggering antigen, appears to be a condition characterized by persistent and disturbing symptoms such as, but not limited to, sleep deprivation and disorders, and it can potentially lead to mental disorders such as depression, anxiety attacks, and somatic disorders, to name a few [17]. Although CSU is a mainly mast cell-driven disease, eosinophils play a significant role in its pathogenesis, acting synergistically and enhancing each other’s actions. Therefore, CSU may be a promising target for IL-5 inhibition because it is a mediator of eosinophil activities such as chemotaxis, differentiation, and survival [18]. Unlike AD, where the results of anti-IL-5 therapy were unsatisfactory, benralizumab—an IL-5R antagonist—was effective in some smaller studies [19,20]. However, due to some cases in which anti-IL-5 therapy exacerbated the symptoms of the disease [21] or no noticeable differences were observed in CSU controls [19], further research is needed before it can be considered a viable treatment option.

Chronic rhinosinusitis (CRS) is another condition proposed for investigation, despite the main line of treatment including nasal irrigation, corticosteroids, and antibiotics that can usually alleviate or even get rid of symptoms. However, some cases have been reported where conventional treatments are not satisfactory [22], and in these situations, a new treatment, potentially anti-IL-5, could improve outcomes, with some studies showing reduced polyp formation and less need for surgical interventions [22,23].

The aim of this review was to analyze in detail the structure, function, and characteristics of IL-5 and then to collect, evaluate, and summarize the current knowledge on the role of IL-5 in the pathogenesis and potential therapeutic targets in several selected diseases with an allergic component such as asthma, AD, CSU, and CRS.

## 2. Methodology

The research was based on the methodology of the Preferred Reporting Items for Systematic Reviews and Meta-Analyses (PRISMA). Electronic database searches focused on original articles, meta-analyses, and systematic reviews regarding IL-5, its role in the pathological pathways of allergic diseases, as well as anti-IL-5 drugs and their effectiveness and applications.

In this study, the databases were searched using PubMed. The research included a review of work from the last 10 years up to May 2024. The keywords included terms such as Interleukin-5, IL-5, Asthma, Atopic dermatitis, Urticaria, Chronic rhinosinusitis Allergy Biological treatment; Benralizumab; Mepolizumab; Reslizumab. The keywords were used both in combination and separately.

Moreover, articles cited in the obtained publications, articles related to the topics discussed, and important publications from the past were analyzed and included. Articles in languages other than English, not directly related to the topic described, and outdated or too limited were excluded.

## 3. IL-5 and Its Receptor

### 3.1. IL-5 Structure

The IL-5 glycoproteins in mice and humans form disulfide-linked homodimers with a molecular mass ranging from 50 to 60 kilodaltons (kDa) [24]. The diversity in IL-5’s molecular weight primarily stems from the variable attachment of carbohydrates during post-translational modifications; however, its N-linked glycoside group probably does not impact its activity [25]. Each chain of the homodimer consists of 115 amino acid residues and exhibits a cytokine fold typical for other cytokines such as IL-2 or IL-4 [26]. What differentiates IL-5 from other interleukins are the symmetrical intermolecular disulfide links. IL-5 is a member of the short-chain helical-bundle subfamily of cytokines [27].

**Figure 1:**
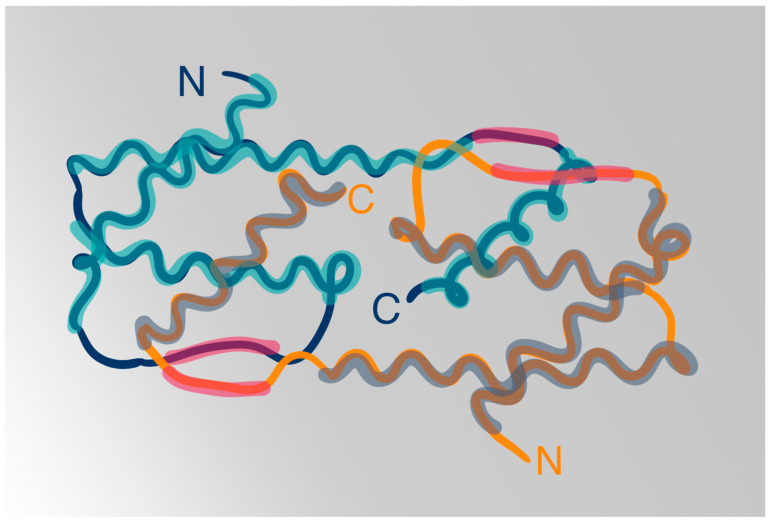
IL-5 particle homodimer structure. Each of the two polypeptide chains consists of an alpha-helical motif beginning at the C terminus. This is followed by a B sheet, alpha-helical, another B sheet, and two other alpha-helical motifs in the N terminus [26,28].

At the center of IL-5 lay two bundles of four left-handed α helices, positioned end to end, and two short antiparallel β strands situated on opposing sides of the dimer [26]. Other proteins within the hematopoietic cytokine family, including IL-3 and granulocyte-macrophage colony-stimulating factor (GM-CSF), also share this common structural feature characterized by the presence of four α helices [28]. Starting at the N-terminus the single polypeptide of IL-5 contains three tightly packed α helices and one β strand [26]. Residues spanning from Cys 86 to the C-terminus constitute the second β strand and a helix, which are packed against the other monomer chain to finalize a bundle comprising four α helices in the up-up-down-down topology and a two-stranded antiparallel β-sheets [26]. The three helices located at the N-terminal side of each polypeptide interact with the C-terminal end and helix of the other polypeptide [29] [Figure 1].

The dimerization mechanism of IL-5 implies a plethora of covalent, electrostatic, and hydrophobic interactions between the two monomer chains, which, while determining its tertiary structure, is necessary for biological activity [2]. The IL-5 receptor binding site is indicated to be located at the C-terminus of each monomer [30]. The IL-5 structure is believed to provide a distinctive functional motif for attaching to its designated receptor subunit, α, and a distinct motif for binding to the “signaling” subunit, βc, of the receptor.

The genetic structures of both mouse and human IL-5 genes comprise four exons and three introns [31]. The degree of similarity between the exons of mouse and human IL-5 genes falls within the range of 70–85% [31]. The amino-acid sequence homology of human and mouse IL-5 is 70% [30].

Eosinophils, mast cells (MCs), basophils, Th2 cells, group 2 innate lymphoid cells (ILC2), invariant natural killer T cells (iNKT cells), and themselves are prominent cellular origins of IL-5 [32].

### 3.2. IL-5 Receptors

IL-5R is expressed in eosinophils, basophils, and mast cells CD34+ progenitor cells [2,28,33]. It can also be found in airway smooth muscle cells [34].

The IL-5R comprises two chains: the IL-5-specific receptor α (IL-5Rα) chain (60 kDa), also known as Cluster of Differentiation 125 (CD125), and a common β chain (βc) (130 kDa, CD131), structurally identical with IL-3 and granulocyte-GM-CSF—all three cytokines demonstrate shared functions in the regulation of hematopoietic cells and are implicated in inducible hematopoiesis [35,36]. βc is sometimes referred to as “Cytokine Receptor β-Common Subunit” [37].

IL-5Rα is a type I transmembrane protein with an NH2-terminal hydrophobic region, a glycosylated extracellular domain containing two pairs of cysteine residues and the WSxWS motifs, a transmembrane domain, and a cytoplasmic domain rich in proline residues [38].

βc forms a homodimer where each chain consists of four domains—the two βc subunits making up the homodimer are intertwined through the exchange of β-strands between domain 1 of one subunit and domain 3 of the other subunit. The interface between domains 1 and 4 in this distinct structure constitutes the functional epitope [36].

Both α and βc subunits contain extracellular fibronectin-III domains, which are occasionally referred to as cytokine recognition motifs [28]. IL-5Rα is responsible for the specific binding of IL-5, whereas βc serves as the signaling unit for further transduction [39,40].

**Figure 2:**
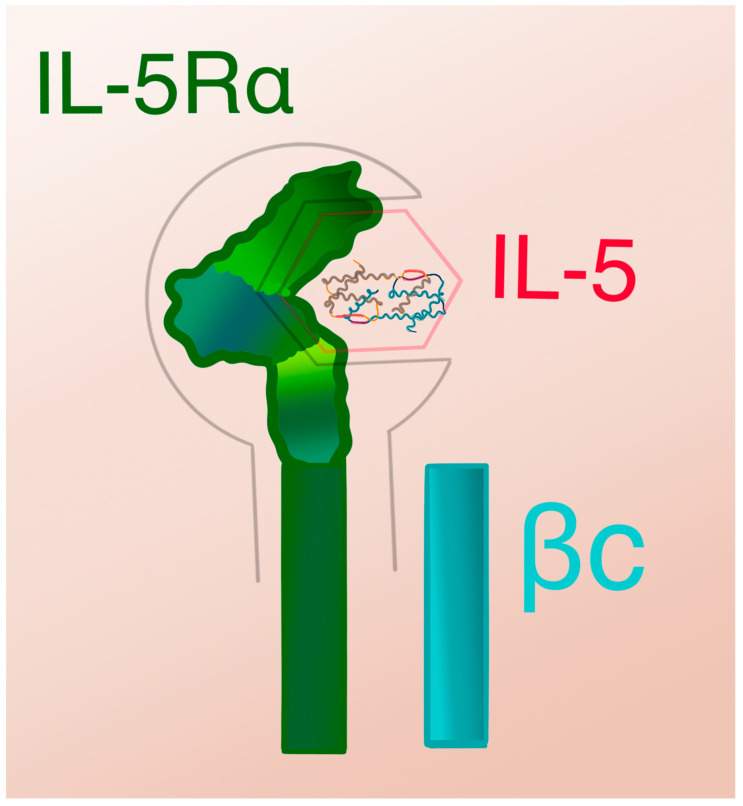
IL-5 receptor alpha subunit forming a “wrench-like” structure with three consecutive domains that allow for specific IL-5 homodimer binding [42].

The analysis of the interaction between IL-5 and its receptor revealed that preformed homo-oligomers of βc subunits assemble with IL-5Rα in the presence of IL-5, which leads to higher-order rearrangements of βc homo-oligomers and an increase in IL-5Rα affinity for IL-5 [35,41]. Therefore, IL-5 induces cellular responses through a two-step mechanism involving the binding of the cytokine ligand to the specificity receptor IL-5Rα, followed by the binding of the ligand-subunit α complex to, and subsequent rearrangement of, a basal form of βc oligomers [28,41]. The binding of IL-5 to its receptor entails dynamic changes in the conformation of binding sites, which are unique to the IL-5 cytokine as well as to the IL-5Rα and βc subunits, with amino acid residues, their positions within epitopes, and charges playing crucial roles [28]. The IL-5Rα surrounds the IL-5 homodimer with three consecutive domains, forming a “wrench-like” structure, which leads to the formation of an octameric motif of IL-5/IL-5RAβ complexes [42] [Figure 2].

### 3.3. IL-5 Signal Transduction

The subunits of the IL-5 receptor lack kinase domains in their cytoplasmic tails. Consequently, the initiation of the signaling cascade relies on the phosphorylation of multiple kinases, including Janus kinase 1 (JAK1) and 2, Src-homology 2 (SH2)/SH3-bearing signaling proteins such as Vav, Src-homology-domain-containing transforming protein (Shc), Bruton tyrosine kinase (Btk), Lyn kinase, p38, phosphoinositide 3-kinase (PI3K), mitogen-activated protein kinases (MAPK), hematopoietic lineage cell-specific protein 1 (HS1), and signal transducer and activator of transcription (STAT) 1, -3, and –5 [39,43,44].

Phosphorylation of these proteins activates downstream molecules such as proto-oncogene serine/threonine-protein kinase-1 (Pim-1), c-fos, and c-jun, as well as nuclear factor kappa B (NFκB), thereby promoting cell survival, proliferation, and immune responses, ultimately influencing the number of eosinophils [43].

Upon binding of IL-5 to IL-5Rα, a sequential activation process is initiated, beginning with the activation of JAK2 and subsequently leading to the activation of STAT1, STAT3, and STAT5 [45]. JAK2 is constitutively associated with IL-5Ra, while JAK1 is constitutively associated with the βc chain [28]. The dimerization of IL-5R subunits triggers JAK2 activation and phosphorylation of the beta subunit even in the absence of JAK1 activation—the tyrosine phosphorylation of JAK1 relies on the prior activation of JAK2 [46]. The subsequently activated STAT proteins then facilitate the transcriptional activities of numerous genes involved in eosinophil proliferation, such as pim-1 and cyclin D3 [45]. This pathway is essential for mediating IL-5-induced effects in both eosinophils and B cells [44].

Furthermore, JAK2 cooperates with both Raf-1 and Lyn kinases, and these interactions result in the inhibition of eosinophil apoptosis similar to Pim-1’s activity and nuclear factor kappa-light-chain-enhancer of activated B cells (NF-κB)-mediated induction of BCL-XL [39,45,47,48]. Moreover, Raf-1 kinase plays a crucial role in eosinophil activation [47].

**Figure 3:**
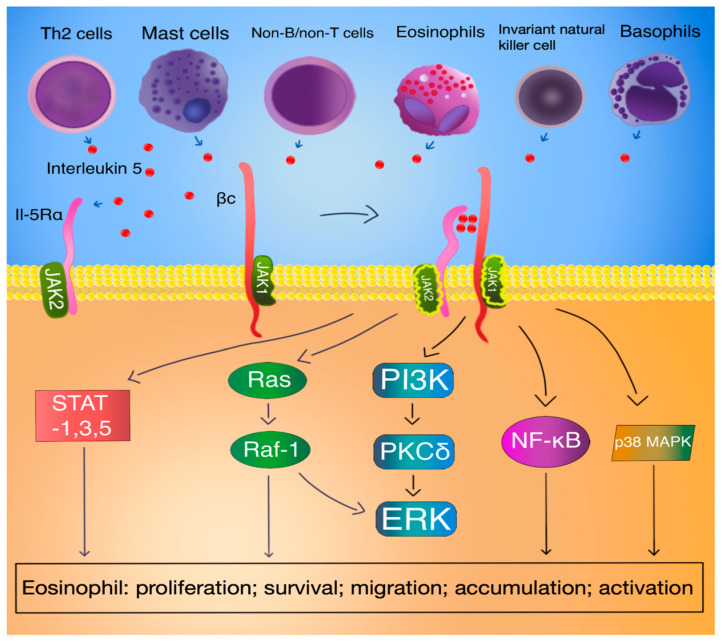
IL-5 signal transduction and functions in eosinophils. The binding of the IL-5 molecule with the IL-5Ra receptor and subsequent dimerization with Bc JAK1 along with JAK2 activation leads to the transduction of multiple intracellular pathways. This significantly impacts eosinophil physiology [28,43,44,46].

Through activation of class IA PI3k, extracellular signal-regulated kinases (ERK), and the protein kinase C δ (PKCδ) pathway, IL-5 induces eosinophil adhesion to endothelial β2-integrin [49]. The IL-5-induced phosphorylation of ERK1/2 is vital for eosinophil degranulation and cytokine production, whereas the activation of p38 is crucial for eosinophil differentiation, degranulation, and cytokine production [50]. Through phosphorylation of p38 and nucleus translocation of NF-κB in eosinophils, IL-5, along with IL-3 and GM-CSF, regulates the chemotaxis and adhesion of human eosinophils [51] [Figure 3].

Moreover, IL-5 prompts the activation of SHP2 and Raf-1 proteins. Particularly, Raf-1 assumes a regulatory function in governing the process of degranulation [43]. The Ras/Raf/MEK/ERK pathway governs the functionality of numerous proteins implicated in apoptosis and links signals received by cell membrane receptors with transcription factors, which oversee the control of gene expression [52]. Such a gene is c-fos, which plays an important role in eosinophil maturation, proliferation, survival, and production of the eosinophil chemoattractant leukotriene C4 [40]. Furthermore, the activation of Ras GTPase-ERK is essential for promoting cell survival and proliferation in response to IL-5 signaling [53].

IL-5Rα can manifest in either a membrane-anchored (TM) active form or a soluble (SOL) variant with antagonistic properties in vitro [54]. Genomic data has uncovered that the synthesis of the TM IL-5R form necessitates alternative splicing in which a soluble-isoform-specific exon is skipped. It was suggested that the two soluble variants of the IL-5 receptor alpha subunit originate either from a “normal” splicing event or from the absence of splicing altogether. In contrast, indeed, the signaling-competent TM isoforms of IL-5Rα are produced through a mechanism of alternative gene splicing [54,55]. This process involves differential switching to exclude exon 11 and include exons 12, 13, and 14, resulting in TM-IL-5Rα. Conversely, inclusion and termination at exon 11 were shown to generate SOL-IL-5Rα [56].

The SOL IL-5Rα exhibits antagonistic properties in eosinophil proliferation and differentiation in vitro; however, such properties may not be exhibited in vivo [54]. It can act as a competitive inhibitor by binding to IL-5, thereby preventing its interaction with the membrane-bound IL-5 receptor and inhibiting downstream signaling events [57]. SOL-IL-5Rα captures IL-5 and creates a complex that does not activate membrane-bound beta chains [55].

Interestingly, another hypothesis for the alternative generation of SOL-IL-5Rα emerged, where IL-5 regulates IL-5 receptors through the proteolytic release of IL-5Rα. This was supported by a dose-dependent reduction in IL-5Rα observed concomitant with an elevation in IL-5Rα levels and a decrease in IL-5-mediated loss of IL-5Rα while matrix metalloproteinase-specific inhibitors were introduced [58]. This down-regulation of IL-5Rα and the subsequent diminished IL-5 response in mature eosinophils may be regarded as a negative feedback loop [59].

IL-5 suppresses the DNA fragmentation and apoptosis of mature eosinophils, which are dependent on the synthesis of new RNA and proteins. Both human and murine mature eosinophils sustain their viability in the presence of IL-5 [60].

Lyn, Jak2, and Raf-1 kinases play crucial roles in the IL-5-induced inhibition of eosinophil apoptosis. While Lyn and Jak2 tyrosine kinases are not essential for the secretion of eosinophil cationic protein and the upregulation of CD11b adhesion molecules, Raf-1 kinase is pivotal for these processes [47].

### 3.4. IL-5 Function

Th2 cells, mast cells, invariant natural killer (NK) T cells, non-B/non-T cells, CD34+ progenitor cells, eosinophils, and basophils are all cellular origins of IL-5 secretion [28].

Th2 cells produce and release IL-5 following an activation process initiated by inhaled allergens and orchestrated by dendritic cells [61].

IL-5 has gained the title of the most important eosinophilopoietic cytokine [62].

IL-33 precedes IL-5 in regulating eosinophil commitment and is essential for eosinophil homeostasis [63]. Treatment of bone marrow cells with IL-33 in vitro results in a specific early proliferation of precursor cells expressing IL-5Rα, while IL-5 does not exhibit this effect. IL-33 supports eosinophilopoiesis by stimulating both systemic IL-5 production and the expansion of precursor cells expressing IL-5Rα [63]. IL-5 promotes eosinophil maturation from these cells [64].

In a homeostatic state, mice lacking IL-5 still have mature eosinophils present in their bone marrow and peripheral blood, albeit in lower quantities, and exhibit decreased numbers of B-1 cells in peritoneal washouts and reduced levels of IgA-producing B-1 cells in the lamina propria [28].

IL-5 plays various roles in eosinophil biology. It is implicated in the differentiation and maturation of eosinophils within the bone marrow, potentially forming a readily mobilizable pool of cells in response to allergens. Additionally, IL-5 facilitates the migration of eosinophils to tissue sites, typically in the context of allergic reactions. Furthermore, it plays a role in preventing eosinophil apoptosis. What is more, IL-5 likely contributes to the capacity of eosinophilic precursors to proliferate, differentiate, and function autonomously within various tissues, including the lungs [28] [Figure 3].

The recruitment of eosinophils from blood microvessels involves IL-5, which facilitates this process by stimulating the release of a rapidly mobilizable pool of eosinophils from the bone marrow into circulation [65] [Figure 3]

Following the allergen challenge, there is a notable rise in the percentage of CD34+ cells that express IL-5Rα in comparison to the levels observed in the bone marrow before allergen exposure, which is followed by blood and sputum eosinophilia. Heightened expression of IL-5Rα on CD34+ cells promotes eosinophil production, potentially contributing to the subsequent emergence of eosinophilia in both blood and tissues [66].

Systemic IL-5 administration leads to an increase in circulating eosinophil progenitors, indicating a role in eosinophil mobilization [67]. Interestingly, enforced retroviral expression of IL-5Rα in granulocyte/monocyte progenitors does not lead to increased progression to eosinophils in the presence of IL-5; however, bone marrow cells of the natural phenotype IL-5Rα(+)CD34(+)c-Kit(lo) develop exclusively into eosinophils, which suggests that IL-5Rα is expressed in cells already pre-committed to the eosinophil lineage [68]. Such early progenitors would be predestined to develop IL-5R at later stages of development, which therefore would not require IL-5 stimulation to occur. Indeed, myeloid line progenitors committed to determined lineages in transcriptionally primed profiles were proven to be misclassified as undifferentiated due to a lack of expression of surface markers [69]. Clusters of phenotypic granulocyte-macrophage progenitors (GMPs) expressing eosinophil-specific genes (IL-5Rα, Epx, Prg2) have been discovered, suggesting that cell type-specific transcription factor networks are either primed or expressed in early eosinophil progenitors [70]. This suggests that defining eosinophile progenitor simply as “CD34+ cells that co-express IL-5Rα on their surface” [71] may be obsolete.

Current research indicates that IL-5 may not be specifically necessary for the commitment to the eosinophil lineage [72]. The initial differentiation of eosinophils is stimulated by IL-3 and GM-CSF, independently of IL-5. However, at the subsequent stages marked by the production of eosinophil peroxidase, IL-5 can serve as the sole growth factor for further development [73].

The impact of IL-5 on the differentiation of eosinophils was found to have another aspect. It was proven that eosinophils differentiate locally from progenitors outside of bone marrow in a highly IL-5-dependent fashion; moreover, this process might be regulated in vivo by endogenous production of soluble IL-5Rα, antagonizing IL-5 function in vitro [74].

It is hypothesized that eosinophils can regulate their sensitivity to IL-5 through controlled expression of such an isoform of the IL-5R. Interestingly, mature eosinophils circulating in the bloodstream predominantly express the SOL IL-5Rα transcript [54].

Progenitor cells, while activated, secrete TH2-like cytokines such as IL-5 and can function as proinflammatory effectors independently, directly contributing to allergic inflammation [75].

It was found that the presence of IL-5 enhances thymic stromal lymphopoietin-dependent eosinophilopoesis in peripheral tissues via increased expression of transcription factors key to eosinophil development—GATA-2 and C/EBPα [62].

Additionally, IL-5 induces the terminal maturation of eosinophils by upregulating C-C chemokine receptor type 3 (CCR3) gene expression, potentially influencing CCR3-dependent chemotaxis by both eosinophils and lymphocytes [67]. CCR3 is a transmembrane G protein-coupled receptor primarily expressed in eosinophils. Unlike most chemokines, which bind to multiple receptors, eotaxins specifically signal only through the CCR3 receptor, the main chemokine receptor responsible for the recruitment of eosinophils to inflamed tissues [71].

Increasing expression of CCR3 chemokine as well as integrin CD11b and IL-5 may facilitate the migration of mature eosinophils from the blood to the airway [47,67]. Nevertheless, IL-4, along with IL-13, are known to play a major role in regulating the transmigration and accumulation of eosinophils in allergic inflammation via increases in P-selectin expression [76].

IL-5 can selectively enhance the chemotactic response of eosinophils [77]. However, despite an observed decrease in bronchial mucosal eosinophil-lineage cells after administration of an anti-IL-5 monoclonal antibody in asthmatic individuals, eosinophils could reside in tissue to some extent [78]. This indicates that, despite IL-5’s important role in eosinophil chemotaxis, it is not a necessary factor.

In addition to their involvement in Th2 responses, eosinophils also play crucial roles in maintaining homeostasis, challenging the traditional view of them solely as inflammatory cells [79]. Under normal conditions, eosinophils quickly exit the bloodstream to enter various tissues, where they regulate diverse biological functions, such as immunoregulation. Certain subsets of eosinophils residing in the lungs exhibit anti-inflammatory and anti-allergic properties and appear to be partially independent of IL-5 signaling, at least in mice [79].

Interestingly, due to the presence of the IL-5 receptor on airway smooth muscle cells, IL-5 can mediate hyperresponsiveness independently of eosinophil activity in vitro [34].

IL-5, along with IL-3 and GM-CSF, amplifies effector capabilities, including chemotaxis and exocytosis in basophils, while also preparing for C5a-induced lipid mediator production [80]. IL-5 does not directly influence histamine release or degranulation itself but instead acts as a facilitator for these characteristic basophil functions [28]. The IL-5 receptor is expressed on basophils to a lesser extent than on eosinophils [28].

Despite IL-5’s significant impact on various eosinophil functions, it was originally discovered as a factor differentiating murine B lymphocytes into IgM-secreting cells [2]. IL-5 impact on lymphocytes embraces the ability to stimulate the production of antigen-specific IgA from B-2 B cells that have been primed with antigen and the induction of the production of polyclonal IgA from B-2 B cells that have been stimulated with lipopolysaccharide [2]. A relationship links IL-5 and ILC2s [81]—cells residing at mucosal regions where they control immunity and are responsible for producing cytokines such as IL-5 and IL-13 [82]. ILC2s play a crucial role in immune defense against helminth infections and contribute to the pathogenesis of airway hyperreactivity [82]. IL-33 stimulation of ILC2s results in the production of high levels of IL-5, ILC2s proliferation, and eosinophil infiltration in the lung; also, IL-5-producing ILC2s may contribute to IgA production [2].

## 4. Biological Treatment

The demonstration of a strong association between eosinophils’ life cycle and IL-5 and their involvement in the pathobiology of eosinophil-driven disorders shifted attention to this cytokine. This, in turn, led to the development of innovative medications that directly target IL-5Rα [83,84,85].

**Figure 4:**
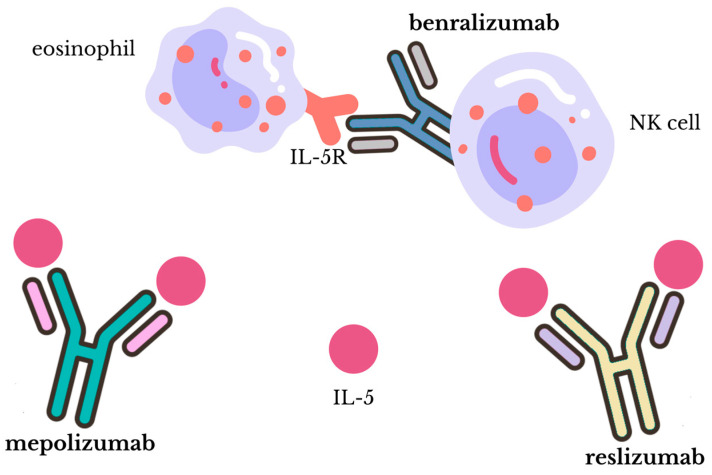
The mechanism of action of mepolizumab, reslizumab, and benralizumab. Mepolizumab and reslizumab both act by binding to IL-5 and preventing its linkage to its receptor, while benralizumab binds to IL-5R and interferes with IL-5 interaction with eosinophils [40].

There are currently three monoclonal antibodies registered: mepolizumab (available under the trade name Nucala™ from GlaxoSmithKline), reslizumab (Cinqair™ or Cinqaero™ from Teva) and benralizumab (Fasenra™ from AstraZeneca). The main difference is in the mechanism of action: mepolizumab and reslizumab target circulating IL-5, while benralizumab works by binding to IL-5Rα present on eosinophils and basophils. These distinct mechanisms contribute to their unique therapeutic properties and potential applications [84,86,87] [Figure 4]. A comparative overview of these three monoclonal antibodies is presented in the table below [Table 1], including aspects such as mechanism of action, use cases, and side effects. A more detailed look at these drugs is then provided.

Mepolizumab (Nucala™, GlaxoSmithKline) is a fully humanized monoclonal N-glycosylated IgG1/k antibody. By binding the α-chain of free IL-5, it prevents IL-5 from associating with the α subunit of the IL-5 receptor on the surface of eosinophils and blocks its actions [83,84,88]. It was found that mepolizumab not only lowers blood and sputum eosinophil counts but also reduces the terminal differentiation of eosinophils in the bone marrow.

The mepolizumab half-maximal effective concentration (EC50) is 0.94 nM, and its dissociation constant (Kd) is 100 pM. The pharmacokinetics of mepolizumab were assessed in clinical trials using doses ranging from 0.05 to 10 mg/kg, as well as fixed doses of 250 mg, 750 mg, and 1500 mg. It exhibited slow elimination kinetics, characterized by mean initial and terminal phase half-life values of approximately 2 and 20 days, respectively. The steady-state volume of distribution ranged from 49 to 93 mL/kg, while plasma clearance varied between 0.064 and 0.163 mL/h/kg [85,88].

Clinical trials were carried out to evaluate the effectiveness of mepolizumab in treating various eosinophil-driven disorders, including severe asthma, atopic dermatitis, nasal polyposis, eosinophilic esophagitis, hypereosinophilic syndromes (HES), eosinophilic granulomatosis with polyangiitis (EGPA), and chronic obstructive pulmonary disease (COPD) [84,88,89,90,91].

Currently, both in the United States and in Europe, mepolizumab is registered for the treatment of severe asthma with an eosinophilic phenotype, chronic rhinosinusitis with nasal polyps (CRSwNP), eosinophilic granulomatosis with polyangiitis, and hypereosinophilic syndrome.

For severe eosinophilic asthma, adults and adolescents aged 12 years and older are recommended a dose of 100 mg administered every 4 weeks by subcutaneous injection into the upper arm, thigh, or abdomen. In children aged 6 to 11 years, a smaller dose of 40 mg is advised over the same interval. This treatment plan is designed as an add-on maintenance therapy for long-term management of the condition, with annual reviews to decide the necessity of continuing the treatment based on disease severity and control of exacerbations.

For CRSwNP in adult patients 18 years of age and older with an inadequate response to nasal corticosteroids, the recommended dose is also 100 mg given subcutaneously every 4 weeks. If no response is observed after 24 weeks, alternative treatments may be considered. However, patients showing partial initial responses may benefit from prolonged therapy beyond 24 weeks. Analogous to that in severe eosinophilic asthma, mepolizumab is also indicated for the add-on maintenance treatment of CRSwNP.

The European Medicines Agency Committee for Medicinal Products for Human Use has recommended administering mepolizumab at a dosage of 300 mg every 4 weeks for the treatment of EGPA in adults and adolescents aged 12 years and older. For younger children aged 6 to 11 years, the dose varies based on body weight: 200 mg for those weighing ≥ 40 kg and 100 mg for those under 40 kg, administered every four weeks. The U.S. Food and Drug Administration has approved the use of Nucala (mepolizumab) for the treatment of EGPA, but only in adult patients, at the recommended dosage of 300 mg every 4 weeks. Like the other conditions, EGPA treatment is aimed at long-term management with annual evaluations to assess the necessity for ongoing therapy.

Similarly, in HES, adults are prescribed a dose of 300 mg every 4 weeks. The approach to long-term treatment and annual review of therapy necessity closely resembles that of EGPA [92,93].

The drugs commonly reported adverse effects encompass infections of the lower respiratory tract and urinary tract, pharyngitis, nasopharyngitis, arthralgia, arrhythmias, hypersensitivity reactions, headaches, eczema, upper abdominal pain, muscle spasms, back pain, and injection-site reactions. Anaphylaxis is a rare occurrence [92,93].

Reslizumab (Cinqair™in USA/Cinqaero™ in Europe, Teva Respiratory, LLC) is an IgG4/k humanized monoclonal antibody. It functions by neutralizing circulating IL-5 and preventing its binding to IL-5 receptor α, which is expressed in various cells, including eosinophils. This action not only disrupts IL-5 maturation and activation but also decreases the survival of eosinophils and consequently lowers the levels of eosinophils in the airways [84,94,95]. Formerly known as SCH-55700, reslizumab was developed by grafting complementarity-determining regions from the rat monoclonal IgG2a antibody JES1-39D10, to create a humanized IgG4/κ antibody with high affinity for human IL-5.

Reslizumab showcases potent inhibitory effects on the growth of the human erythroleukemic cell line TF-1 stimulated by IL-5, with an EC50 value of 45 pM. Additionally, it demonstrates a Kd of 81 pM [96,97].

Pharmacokinetically, reslizumab reveals a distribution volume of around 5 L, clearance of approximately 7 mL/hour, and a half-life of about 24 days [98].

The clinical research on reslizumab has encompassed a wide range of conditions, including asthma, eosinophilic granulomatosis with polyangiitis, eosinophilic esophagitis, and hypereosinophilia following diethylcarbamazine treatment of Loa-Loa infection. This extensive scope underscores the potential versatility of reslizumab in addressing eosinophil-associated pathologies [84,99].

In 2016, the European Medicines Agency and the U.S. Food and Drug Administration accepted reslizumab as an additional treatment in patients with severe asthma with an eosinophilic phenotype for patients aged 18 years and older with severe asthma displaying an eosinophilic phenotype unresponsive to high-dose inhaled corticosteroids in combination with another asthma preventive medication. In Europe, dosing guidelines vary significantly based on the patient’s body weight. For patients weighing less than 35 kg or more than 199 kg, the dosing recommendation is set at 3 mg/kg of body weight. For patients within the weight range of 35 kg to 199 kg, the dosing follows a predefined vial-based scheme, and it varies between 100 mg and 575 mg. In both cases, reslizumab is administered via intravenous infusion once every four weeks.

In the USA, the method of administering the drug is identical to that in Europe, but the doses do not vary depending on the patient’s weight and are administered at a standard of 3 mg/kg once every 4 weeks [98,100].

The most frequently reported adverse events encompass myalgia, nasopharyngitis, worsening of asthma symptoms, upper respiratory tract infections, and increased blood creatine phosphokinase. Notably, the incidence of the serious adverse reaction of anaphylaxis was reported in 0.2% of asthma patients in placebo-controlled clinical studies [84,98,100].

Benralizumab (Fasenra™, AstraZeneca) represents a distinct advancement in biological therapies due to its unique mechanisms of action and target specificity. As a fully humanized, afucosylated monoclonal antibody, it selectively targets IL-5Rα, which is found not only in human eosinophils but also in basophils. This binding not only inhibits the activation of IL-5Rα but also triggers apoptosis of these cells via antibody-dependent cell-mediated cytotoxicity (ADCC), facilitated by its high affinity for the human FcγRIIIa receptor. This enhanced ADCC capability leads to a significant reduction in levels of circulating eosinophils and basophils, widening its therapeutic potential [10,84,86,101,102].

Benralizumab binds with high affinity to the extracellular domain of IL-5Rα, with a Kd of 11 pM. It stained human peripheral blood eosinophils with an EC50 of 26 pM. Pharmacokinetically, benralizumab is characterized by a central volume of distribution of 3.1 L and a peripheral volume of distribution of 2.5 L in a standard 70 kg individual. The drug follows linear pharmacokinetics. Notably, there is no evidence of receptor-mediated clearance. The estimated systemic clearance rate for benralizumab is 0.29 L per day. After subcutaneous administration, benralizumab exhibits an elimination half-life of approximately 15.5 days [103,104,105].

Approved in the USA since 2017 and in Europe in 2018, benralizumab is employed notably as an add-on maintenance treatment for adult patients with severe eosinophilic asthma who are inadequately controlled with high-dose inhaled corticosteroids and long-acting β-agonists. In the USA, however, the target group also includes younger patients, starting at 12 years of age.

The standard dosing regimen begins with 30 mg administered by subcutaneous injection every 4 weeks for the first three doses, followed by a maintenance dose every 8 weeks thereafter [104,105].

The most common adverse events associated with benralizumab reported in clinical trials include pharyngitis, hypersensitivity reactions, anaphylaxis, headaches, pyrexia, injection-site reactions, and nausea [84,102,104,105]

The exploration of benralizumab extends beyond eosinophilic asthma to potentially treat other inflammatory conditions like COPD, HES, and CRS, indicating its broad application in immunological therapy [84,102].

## 5. Eosinophilic Asthma

### 5.1. Asthma–Basic Facts

Asthma is a heterogeneous lung condition that affects the airways, and its symptoms are, among others: wheezing, shortness of breath, chest tightness, and cough. They are the result of airway hyperresponsiveness and chronic inflammation leading to the contraction of smooth muscles and swelling of the bronchial mucosa, the formation of mucus plugs, and, over time, also remodeling of the bronchial wall. The definition of asthma also includes the variability of the previously mentioned symptoms over time. Its course includes periods of both exacerbations and remissions. Exacerbations can be triggered by several factors, such as allergens, infections of the respiratory tract, smoking, physical exercise, irritants, emotional reactions, or medication [106,107].

The diagnosis of asthma is made using the Global Initiative for Asthma (GINA) criteria. Lung function tests, such as spirometry or measuring the peak expiratory flow (PEF), need to be performed. In spirometry, the most important parameters are forced expiratory volume in the first second (FEV1) and FEV1 to forced vital capacity (FEV1/FVC). What is worth emphasizing is that, apart from basic spirometry, diastolic test and bronchial provocation test results need to be assessed. In adults with typical respiratory symptoms, an increase or decrease in FEV1 of >12% and >200 mL or PEF of >20% allows for the diagnosis of asthma [106].

The therapy of asthma is based on controller medications, such as inhaled corticosteroids (ICS), Long-Acting Beta-Agonists (LABA), Long-Acting Muscarinic Antagonists (LAMA), and reliever medications, including Short-Acting Beta Agonists (SABA) [106].

The pathophysiology of eosinophilic asthma is inextricably linked with IgE antibodies that respond to certain triggers and then bind to high-affinity mast cells and basophils. The mast cells then release histamine, prostaglandins, and leukotrienes. An integral role is also played by Th2 lymphocytes, which produce a series of interleukins (e.g., IL-4, IL-5, IL-13, IL-25, IL-33) and GM-CSF, which sustain inflammation [108]. IL-4 is responsible for differentiating naive CD4+ T cells into Th2 cells, promoting the secretion of IL-5 and IL-13 and the production of IgE antibodies. IL-4 and IL-13 also play a role in influencing endothelial cell adhesion, while IL-5 is an important factor in eosinophil recruitment, maturation, and activation [109]. Those interleukins also further stimulate the mast cells [107]. All that leads to bronchoconstriction and inflammation and further to intermittent airflow obstruction [108].

### 5.2. IL-5 in Asthma

When it comes to asthma pathogenesis, IL-5 is known to have a pivotal role. In humans, IL-5 acts specifically on eosinophils and basophils by binding the alpha chain of its specific receptor located on those cells. IL-5 is sufficient when it comes to eosinophil maturation, proliferation, activation, and migration, antibody-dependent cytotoxicity, and adhesion to vascular endothelium, as well as prevention of their apoptosis [40,60,110,111,112]. Eosinophil infiltration into the airways after allergen challenge and peripheral blood eosinophilia are observed in atopic asthmatic people. Eosinophil counts in peripheral blood and bronchoalveolar lavage (BAL) fluid are also higher in asthmatics in comparison to healthy controls [111,113].

The activation of eosinophils by IL-5 leads to their degranulation and the release of certain substances such as eosinophil peroxidase (EPO), major basic protein (MBP), leukotrienes, IL-13, and transforming growth factor-β (TGF-β). These mediators, when acting in the respiratory tract, directly or indirectly result in airway hyperresponsiveness, mucus production, airway remodeling, and tissue damage. Those processes are responsible for the pathogenesis and symptoms of asthma [113].

Many studies on both animal and human models of asthma have shown that there is a close correlation between IL-5 and eosinophilic inflammation. It was found that IL-5 promoted airway eosinophilia and bronchial hyperresponsiveness induced by allergen challenge. The increase in IL-5 mRNA levels is associated with the fact that the bone marrow produces more eosinophils in response to antigen challenge [40,62].

Elevated levels of IL-5 can be found in induced sputum from patients with allergic asthma and patients experiencing acute asthma exacerbations, as well as in their serum [40]. It was also found that there is a link between increased levels of IL-5 in serum and BAL fluid and the increased severity of asthma [40,111]. This is why targeting IL-5 and its receptor is a promising course in the treatment of eosinophilic asthma.

### 5.3. Targeting IL-5 in the Treatment of Asthma

When it comes to the treatment of eosinophilic asthma targeted at IL-5, there are currently three drugs available. Those are monoclonal antibodies and include mepolizumab, reslizumab, and benralizumab—the IL-5R antagonist. Mepolizumab is a humanized IgG1/k monoclonal antibody, while reslizumab is a humanized IgG4/κ monoclonal antibody. They both bind to IL-5, which prevents its linkage to its receptor, IL-5R, on eosinophils. Benralizumab, a humanized afucosylated IgG1/κ monoclonal antibody, has a different mechanism of action. Its Fab fragment selectively recognizes domain 1 of human IL-5Rα, near the IL-5 binding site, and interferes with IL-5 binding to eosinophils. Additionally, its Fc fragment binds to the FcγRIIIa membrane receptor on natural killer cells (NK cells), which leads to the release of granzyme B and perforin and further eosinophil apoptosis [40].

All of them are approved and incorporated into current GINA guidelines considering the treatment of difficult-to-treat and severe asthma [114]. Many studies, both clinical and real-life, and meta-analyses have found that treatment targeting IL-5 and its receptor is highly effective and safe [6,7,8,9,10].

When it comes to mepolizumab, it can be administered both intravenously and subcutaneously [115]. It was found that treatment with its use in patients with severe asthma resulted in a significant decrease in blood eosinophil counts, which was consistently lower than in those in the placebo group. It also lowered sputum eosinophil levels. When it comes to lung function, a progressive improvement in lung function parameters, including FEV1, was observed [8,40,86,110,116]. However, there were no statistically significant differences in patients with initial FEV1 values greater than 80% [8]. It was also found that the administration of mepolizumab reduced the rate of asthma exacerbations, including those requiring emergency department treatment [8,40,86,110,116]. In those patients, the use of bursts of oral corticosteroids (OCS) was also observed to decrease [8,40,86,110,116]. Treatment with mepolizumab also improved asthma control, as measured by the Asthma Control Test (ACT) [8,40,110,116]. An important effect is also the improvement in patient health-related quality of life (HRQoL), measured by the St. George’s Respiratory Questionnaire (SGRQ) and Asthma Control Questionnaire (ACQ-5) questionnaires [40,86,110].

Reslizumab can be administered intravenously [115]. Studies showed that reslizumab, as well as mepolizumab, reduced the blood and sputum eosinophil counts. Furthermore, its use was beneficial when it came to lung function—it enhanced FEV1. The reduction of clinically significant asthma exacerbations and improved asthma control were also observed [40,86,110] However, it was observed that reslizumab is more effective when it is used by patients with more severe disease and high eosinophilia [110].

Benralizumab is administered subcutaneously [115]. Studies demonstrated that its administration led to a reduction of the blood levels of eosinophils and that this effect was achieved in a shorter time compared to mepolizumab and reslizumab [8,40,86,110,116]. The lung function, characterized by FEV1, was also improved; however, like with mepolizumab, the difference was significant in patients with initial FEV1 lower than 80% and with eosinophilia [8,86]. Furthermore, the use of benralizumab decreased the number of exacerbations, and the use of OCS improved asthma control, as measured by ACT. It also had a positive impact on HRQoL [8,40,86,110,116].

When it comes to side effects, they include a higher occurrence of pyrexia, headaches, reactions at injection or infusion sites, and hypersensitivity. In some patients, an incomplete therapeutic effect was also observed [117]. However, most studies confirmed that the use of anti-IL-5 therapies is efficient, well tolerated by patients, and safe, also in long-term observations [40,110].

Thus, the use of mepolizumab, reslizumab, and benrolizumab is a promising new treatment option for severe eosinophilic asthma with poor control. There is also a field for further exploration when it comes to predicting the response of patients to those medications or the risk of relapse after withdrawal.

### 5.4. The Role of IL-5 in Asthma-Like, Eosinophilic Diseases

IL-5 plays a significant role when it comes to allergic diseases mediated by eosinophils. The connection of IL-5 with its receptor on eosinophils leads to the activation of immune responses and extracellular matter remodeling and plays a vital role in the maturation and survival of eosinophils [43,118]. Its elevated levels are seen in eosinophilic diseases, not only asthma but also chronic hyperplastic eosinophilic sinusitis, hay fever, eosinophilia, eosinophilic esophagitis, cystitis, fasciitis, eosinophilic granulomatosis with polyangiitis, eosinophilic myocarditis, cellulitis, gastritis, or colitis [43]. Thus, the implementation of anti-IL-5 therapy, already used in asthma, could be beneficial for patients with other eosinophilic diseases and is a promising field for scientists.

## 6. Atopic Dermatitis

Investigation of IL-5 and the conduct of research on immunological pathways in allergic diseases can lead to the assumption that IL-5 is one of the main or initial points in the pathogenesis. Therefore, applications of anti-IL-5 treatment could stop or at least slow down most allergic diseases. As proven above, these trials work great for asthma treatments. Further considerations will include the next one with the highest rate of prevalence worldwide—atopic dermatitis.

AD is described as an inflammatory skin disease where recurrent eczema-like lesions and intense itching are present. As a chronic disease affecting both physical health and mental well-being, it relates to an increased risk of multiple health issues, such as asthma, allergic rhinitis, and mental disorders like depression [12]. According to the Global Report on Atopic Dermatitis 2022, AD affects up to 20% of children and up to 10% of adults [11].

While talking about the theoretical basis, AD is based on the unbalance between lymphocytes Th1 and Th2. At this point, the general rules can be mentioned. The very first sign of the acute disease is a high level of Th2. Additionally, a high level of Th1 is connected with chronic ones. Moreover, the profile of secreted cytokines and interleukins is different in the acute and chronic stages of the disease [12,119,120,121].

AD can be classified into many different subtypes, based on certain biochemical pathways, alternative triggers, and genetic or epigenetic mutations, depending on the relevant variable. Therefore, following the IgE level in the blood, extrinsic AD and intrinsic AD are described [12]. The first one refers to a high level of IgE (>200 kU/L), which is called also allergic AD. The second one is nonallergic AD with a lower IgE level (<200 kU/L). Also, cytokine expression (e.g., IL-5, IL-13, IL-1β) is higher in extrinsic AD than in intrinsic AD. Nevertheless, from the clinical point of view, both of these groups are similar, and symptoms can be equal [119,121,122].

**Figure 5:**
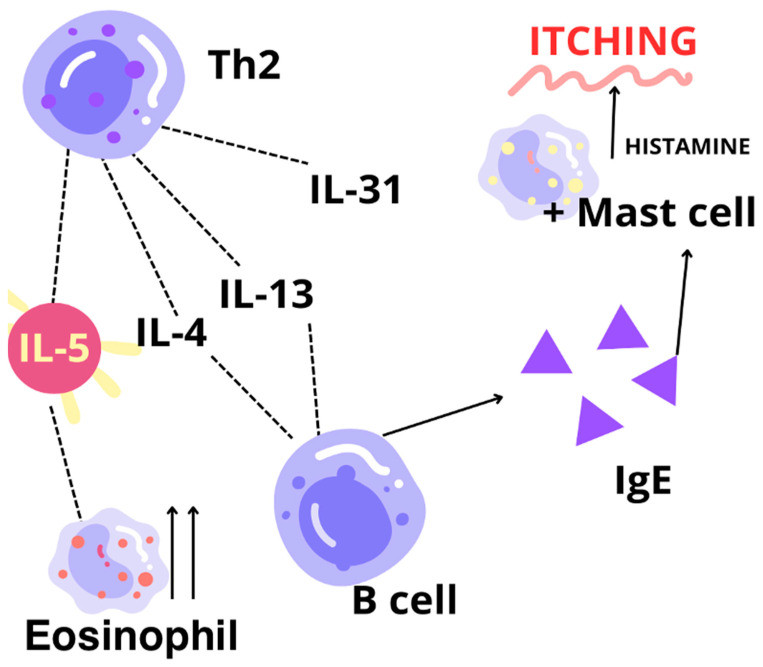
The basic profile of interleukin secretion in AD—even with as efficient an impact on the activity of eosinophils and their migration as IL-5 has, it still seems that the pivotal role in AD is assigned to IL-4 and IL-13 [12,119,120,121].

Going forward, when it comes to the acute phase of AD, there is an early phase of the IgE-dependent immune response [Figure 5]. In this phase, dominated by Th2 lymphocytes, subsequently, an increase in the synthesis of interleukins: IL-2, IL-4, IL-5, IL-6, IL-10, IL-13, IL-21, and IL-31, is observed. Meanwhile, in the chronic phase, being named a late phase with the dominance of Th1, there is a high level of cytokines like IFN-γ, TNF-α, IL-2, IL-8, or IL-12 [121,123,124].

When focusing attention on IL-5 in AD, an increase in IL-5 expression induces the chemotaxis of eosinophils. Moreover, IL-5 extends eosinophil survival time, and what is significant for the clinical observations of patients is that it leads to the initiation and formation of skin inflammation. This can be also observed by using immunohistochemical techniques, where peripheral and cellular eosinophilia are visualized as the layer of proteins derived from degranulated eosinophils [13,119].

While looking at two subtypes of AD with high and lower levels of IgE, IgE is estimated to be increased in 70–80% of patients (Th2-based disease, the extrinsic AD). This IgE-high-level group of patients seems to be a potential one for treatment with anti-IL-5 [119,120,123]. However, even with as effective results as the asthma therapy reveals, the outcome of using anti-IL-5 in AD has remained almost unchanged since the study from 2005 in this field [14,15,16].

In the above-mentioned work, anti-IL-5 recombinant humanized monoclonal antibody (Mepolizumab) for the treatment of atopic dermatitis by J.M. Oldhoff, U. Darsow, and others, the team tested the effectiveness of asthma therapy on AD patients. The idea for this new application for anti-IL-5 was based on the significant role that eosinophils play in allergic diseases. According to the molecular pathways, not only in asthma pathogenesis but also in IgE-dependent AD, the biomolecular mechanisms of IL-5 action are indispensable for eosinophil growth, differentiation, and migration. Therefore, in both illnesses, from the theoretical point of view, anti-IL-5 drugs should stop or at least limit the disease’s progress. However, their study revealed that even though reducing purposefully peripheral blood eosinophil numbers was observed after mepolizumab treatment compared with placebo, zero clinical success was reached [14]. Moreover, no clinical improvement has been seen since [15,16,122,125].

From time to time, attempts are made to apply the mepolizumab therapy to AD. However, the results remained unsatisfactory, not only for researchers but for patients as well. Patients’ reluctance to accept ineffective therapy results in, among other things, resignation from proposed treatment methods [15].

There are some diversified explanations for why anti-IL-5 therapy does not occur as efficiently in AD as for asthma treatment. Although studies show that the peripheral blood eosinophil level slightly decreases after applied treatment, this may not interact the same way with the eosinophil level in skin tissue where the inflammation happens. There was also an idea that the noticeable reduction of tissue eosinophils may take a much longer amount of time, but even longer trials did not turn out to be a success. Additionally, even the reduced amount of blood eosinophils can still migrate to the skin due to chemotaxis. While eosinophils carry CCR3, the combination of therapy with IL-5 antagonists and CCR3 antagonists may be the more effective solution. Lastly, the proper doses and outline of treatment also should be taken into consideration to establish the suitable level of peripheral eosinophil blood level at which the clinical effect may be obtained [13,14,15,123].

Nowadays, lots of attention is given to IL-31, which is suspected to be the main interleukin that causes incessant itching, or to IL-4 and IL-13. Especially in AD, therapy with dupilumab, an antibody against a subunit of the IL-4/13 receptor, brings such good results that it is no wonder that much less effective methods are not widely researched [126]. However, treatment with anti-IL-5 antibodies can be considered as an additional line of treatment in cases of several allergic diseases overlapping, but these studies still need a lot of work and arrangements to define the medical guidelines [122,127,128].

## 7. Chronic Spontaneous Urticaria

CSU is described as a specific form of urticaria in which symptoms like wheals and angioedema, with accompanying pruritus, appear spontaneously without direct connection with the allergen. The duration of symptoms is at least 6 weeks, lasting continuously or sporadically [17]. A series of pruritic wheals tends to disappear within 24 h and recur [129]. CSU usually disappears spontaneously, but it can take up to 5 years for this to happen. When it comes to prevalence, CSU is not as common as asthma or AD, as it applies to approximately 0.5% in Europe and mostly affects women [130]. Because it is not caused by a specific antigen that is potentially easy to imitate and lasts chronically, the effects of CSU are also long-lasting and troublesome, e.g., sleep disturbances, fatigue, overstimulation, and tiredness. It can also lead to some major psychiatric conditions, including depression, anxiety attacks, and somatoform disorders [17].

As already described, CSU is not directly connected with exposure to a specific causative agent. The molecular mechanism leading to CSU is characterized by the presence of specific IgE or IgG autoantibodies. What is very characteristic of this condition is that it involves mast cell activation. As a result of their activation, influx of inflammatory cells, immune cascade, increased inflammatory response, and vasodilatation happen. Additionally, certain medical conditions, such as vitamin D deficiency, infections, and coagulation disorders, also appear to play a role in the etiology of CSU [17].

Recently published new guidelines for CSU do not describe any anti-IL-5 treatment as a form of therapy; from biological drugs, only omalizumab (a monoclonal antibody that selectively binds to human IgE) is mentioned [130]. However, various biologics and other drugs seem to be also considered as potential treatments, as described in clinical trials [17].

Even when CSU is featured mainly as a mast cell-driven disease, the role of eosinophils is emphasized, and it seems to be more crucial in pathogenesis than in the AD featured above. Both mast cells and eosinophils act synergistically as their actions enhance each other. The release of mast cell cytokines develops Th2 inflammation, the type of immune response that involves the recruitment and accelerated influx of eosinophils. Here, it is also worth highlighting that IL-5 plays an important role in this process. Without IL-5, eosinophils are not able to travel from the bone marrow through the tissues and blood barriers to the site of allergic inflammation [18]. Such observations led directly to conclusions about using anti-IL-5 therapy to at least minimize the symptoms of CSU.

While taking inspiration from its pathogenesis, anti-IL-5 drugs would be beneficial in cases of eosinophilic profiles in patients, such as a 27-year-old woman suffering from both asthma and CSU. Because anti-IL-5 therapy is included in EU for adults with eosinophilic phenotype severe asthma as an add-on treatment, she was treated with mepolizumab 100 mg, every 4 weeks. This case is probably the first described, where using anti-IL-5 medications results in the improvement of the urticaria control test (UCT). Additionally, before this therapy, CSU was strongly uncontrolled and had a distressing exacerbation without a clear cause or after any infection. While under this treatment, she reported complete disease control, with no urticarial lesions, even during infections [131]. As this paper was a promising new alternative, more research was started. However, further data are unclear.

While still being reported in some smaller studies as effective [19,20], there are also cases in which the anti-IL-5 therapy exacerbates disease symptoms. With this being considered, it seems that anti-IL-5 therapy (benralizumab in this case) can act bidirectionally, while modifying the symptoms of CSU. Although it mostly works as a blocker of IL-5, it also could act to intensify the CSU symptoms [21].

Moreover, a potent point in the discussion about the validity of anti-IL-5 therapy at CSU is delivered by this year’s study by Sabine Altrichter and others [132]. In their research, a 24-week, randomized, double-blind, placebo-controlled trial with adult patients suffering from CSU was run. The trial divided patients into groups of different benralizumab doses, such as 30 mg, 60 mg, and placebo. As a result, after using benralizumab for 24 weeks, a decrease in the level of eosinophils in the blood was observed. However, no noticeable differences in CSU controls between patients on placebo and patients with benralizumab treatment were observed. This could lead to an observation similar to AD: even if anti-IL-5 therapy decreases effectively the eosinophil’s blood level, there is no statistically significant benefit in this therapy in the case of CSU [132].

Looking at the described research, it seems that anti-IL-5 therapy could be considered in certain situations, but the results remain unclear. This treatment probably will not be considered as a basic therapy line, although more data are needed.

## 8. Chronic Rhinosinusitis

Other quite promising results are received in the case of CRS, described as the inflammation state of the sinonasal mucosa. It is characteristic of CRS to last at least 12 weeks, in which symptoms lower the physical and psychological well-being of patients. CRS can be related to approximately 11% of the general population (both the USA and Europe). From a clinical point of view, two types of CRS can be distinguished—CRSwNP or without them [22,133,134].

The basic treatment line for CRS is based on nasal washes, corticosteroids, and antibiotics, with doses and outlines depending on the intensity of symptoms and exacerbations. In cases of obstructive lesions, functional endoscopic surgery of paranasal cavities is applied. These treatments often work completely or significantly improve the patient’s condition, but some people do not respond satisfactorily. Newer therapies are aimed at patients with treatment-resistant CRS [22].

With time, the clinical division has started to play a less and less important role in planning treatment tactics, and modern practice focuses more on endotype differentiation. The division is based on specific molecular pathways and biomarkers. Moreover, when it comes to CRS biomarkers, the most commonly found biomarker is IL-5, followed by IL-13 and IL-4 [135].

Current guidelines from the European Position Paper on Rhinosinusitis and Nasal Polyps 2020 describe CRS main endotypes as Type 2 and Non-type 2 inflammation. The mentioned Type 2 endotype is associated with CRSwNP and the eosinophilic one (ECRSwNP), depending on eosinophil levels. In ECRSwNP, the tissue eosinophil count ≥10 per high-powered field or blood eosinophils ≥250 cells per microliter. What is worth highlighting is, that Type 2 seems to be the most vulnerable to biological therapies. Therefore, identifying the endotypes helps with treatment selection [135,136].

In these considerations, IL-5 seems to be a possible key mediator of eosinophil actions that play a significant role in Type 2 CRS, especially when eosinophilic CRS is often the basic treatment-resistant one [135,136]. While the IL-5-dependent pathway of eosinophil action, chemotaxis, differentiation, and survival would be inhibited, it could be possible to significantly limit the increasing inflammation, favoring the formation of new inflammatory foci and thus the formation of polyps [22].

From the research conducted so far, the anti-IL-5 treatment seems to decrease the formation and development of nasal polyps in CRS patients [22,134,137,138,139,140]. The data are based on numerous clinical trials and case reports, mostly using benralizumab, that was considered in several meta-analyses, from 2000 to the most recent [22,138,139,141]. Moreover, the applied biological therapy led to a significant decrease in the qualifications of CRSwNP patients for surgical treatment [23]. An additional benefit is an improvement in the sense of smell (83% of patients), which is commonly impaired in CRSwNP [140,142,143]. As an example, the real-life study from last year can be mentioned, describing the application of long-term treatment with omalizumab, mepolizumab, reslizumab, or benralizumab for patients suffering from both severe asthma and CRSwNP. Interestingly, all medications were similarly effective in the improvement of olfaction [143].

What is also noticeable in studies, is the response to anti-IL-5 treatment and the strength of therapy depends on the eosinophils level in patients, and patients with the highest levels obtain the most satisfactory results [144].

The data seem promising, and the proposed therapy can efficiently help with the limitation of the complications of CRS. The therapy risks and side effects also appear to be low and comparable to placebo, which makes this treatment line promising and well-tolerated. A lot of results relate to patients with both asthma and CRS, the certainty of evidence is mostly low, and there is a need to determine the long-term effects of applied therapy [22,139,140]. Luckily, the situation is slowly changing and there are more and more studies proving effectiveness regardless of asthma, as research is conducted on an increasingly larger scale proving the effectiveness of anti-IL-5 therapy, which also could help in the future to limit the use of widely used corticosteroids and to avoid surgery [145,146,147,148].

In one of the most recently published studies by Desrosiers et al. from January 2024 [149], the sustained efficiency of mepolizumab treatment is described. The study was conducted for 52-week plus another 24-week follow-up, allowing us to observe the long-term impact of mepolizumab in CRSwNP. Here, the applied doses were given every 4 weeks, 100 mg subcutaneously. As the results of the 76-week observation, the reduced symptoms, corticosteroid use, and improvement in quality of life were partially observed. These promising results indicate the need to conduct research, that focuses on the durability of mepolizumab therapy in CRSwNP. As the results are mostly promising, more data with a high evidence profile and general official guidelines are needed [22,139,140,146,148].

## 9. Conclusions

In this review, we described in detail the structure, function, and subsequent role of IL-5 in the pathogenesis of various allergic diseases. Furthermore, we summarized the current knowledge on therapeutic applications of IL-5 inhibition in eosinophilic asthma difficult-to-treat, and severe asthma. Although the GINA guidelines include anti-IL-5 and IL-5R drugs, there is room for further research in predicting a patient’s response to a given drug, as well as the risk of relapse after withdrawal.

In AD, several studies have failed to demonstrate the effectiveness of anti-IL-5 treatment using mepolizumab as a test drug. Possible reasons for this were listed, including but not limited to an ineffective reduction of the level of eosinophils in the skin tissue where inflammation occurs, as opposed to the level of eosinophils in the peripheral blood.

Although the new guidelines for the management of CSU do not describe anti-IL-5 treatment methods, there were case studies that suggested the possibility of using this treatment in patients with an eosinophilic profile. However, the data are unclear as there have been reports of CSU exacerbations, questioning the positive effect. Moreover, a randomized, double-blind, placebo-controlled trial did not show statistically significant benefits of this therapy for CSU, thus raising similar concerns as in the case of the treatment of AD.

On the other hand, in CRS, since the most common biomarker is IL-5, the use of anti-IL-5 treatment is more promising. Reduction in the formation and development of nasal polyps in CRS patients, reduction in the qualifications of CRSwNP patients for surgical treatment, and improvement in the sense of smell are the advantages of this treatment, as supported by current data. It is worth noting that the higher the eosinophil level in CRS patients, the better the results of anti-IL-5 treatment. Even though most data come from studies in which patients suffer both from asthma and CRS, there are studies showing the effectiveness of anti-IL-5 treatment regardless of asthma, with patients benefiting from symptom relief, reduced corticosteroid use, and improved quality of life. However, due to the insufficient amount of data, further high-quality studies and formal guidelines are still needed in these cases.

Additionally, in order to organize the collected information and research work, a table was created [Table 2].

## Figures and Tables

**Table 1:** Comparison of registered anti-IL5 monoclonal antibodies.

	Mepolizumab	Reslizumab	Benralizumab
Trade name	Nucala™ by GlaxoSmithKline	Cinqair™ (USA)/Cinqaero™ (EU) by Teva Respiratory	Fasenra™ by AstraZeneca
Mechanism of action	Binds free IL-5, preventing its association with the IL-5 receptor	Neutralizes circulating IL-5, preventing its binding to the IL-5 receptor	Binds to the IL-5Rα on eosinophils and basophils, induces apoptosis via ADCC
Target	Circulating IL-5	Circulating IL-5	IL-5Rα on eosinophils and basophils
Registered use cases	Severe asthma with an eosinophilic phenotype (in children over 6 years of age, adults and adolescents), CRSwNP (adults) EGPA (EU—in children over 6 years of age, adults and adolescents; USA—adults only) and HES (adults)	Severe asthma with an eosinophilic phenotype (adults)	Severe asthma with an eosinophilic phenotype (EU—adults; USA—adults and children over 12 years of age)
Adverse Effects	Lower respiratory tract and urinary tract infections, pharyngitis, nasopharyngitis, arthralgia, arrythmias, hypersensitivity reactions, headache, eczema, upper abdominal pain, muscle spasms, back pain, injection site reactions, anaphylaxis	Myalgia, nasopharyngitis, exacerbation of asthma, upper respiratory tract infections, increased blood creatine phosphokinase, anaphylaxis	Pharyngitis, hypersensitivity reactions, anaphylaxis, headaches, pyrexia, injection-site reactions, and nausea

Abbreviations: ADCC, antibody-dependent cell-mediated cytotoxicity; CRSwNP, chronic rhinosinusitis with nasal polyps; EGPA, eosinophilic granulomatosis with polyangiitis; HES, hypereosinophilic syndrome; IL-5Rα, alpha chain of the interleukin-5 receptor.

**Table 2:** The summary and conclusions were collected based on the paragraphs describing asthma, AD, CSU, and CRS.

Disease Entity	IL-5’s Role	Effectiveness of Anti-IL5 Therapy	Extra Comments
Asthma	Airway eosinophilia and bronchial hyperresponsiveness induced by allergen challenge, mucus production, airway remodeling, and tissue damage [106,110,113]	It was found that therapies targeting IL-5 and its receptor are effective when it comes to the improvement of lung function, decreased eosinophil blood and sputum counts, and reduction of asthma exacerbations [8,40,110,116]	There are studies suggesting that vaccination against IL-5 could be an effective option for treatment [43].
Atopic dermatitis (AD)	Higher expression in extrinsic AD, therefore causing higher Eozynophil level and their migration. However, this is not the most important role in pathogenesis [119,120,121]	It appears to be ineffective in stopping the progression and recurrence of the disease [14,15,16,122,125]	Much better results and effective therapies are directed against IL-31, IL-4, or IL-13 [126]
Chronic Spontaneous Urticaria (CSU)	Both mast cells and eosinophils act synergistically in pathways, where IL-5 plays an important role [18]	The results are uncertain, single case reports confirm the effectiveness of the therapy in patients with a strong eosinophilic profile. However, cases of exacerbations or lack of clinical effect have also been described [19,20,131,132]	So far, only omalizumab is officially used in therapy of CSU [130]
Chronic rhinosinusitis (CRS)	Types of CRS are created based on different endotypes. IL-5 is one of the most common biomarkers in CRS [135]. The IL-5-depended pathway stimulates eosinophil action, chemotaxis, differentiation, and survival, increasing inflammation [22,135,136]	The data seem promising, the therapy can efficiently help with the limitation of the complications of CRS. The therapy risks and side effects also appear to be low [22,139,140].	Initially, most reports were based on patients suffering from both asthma and CSR. Recently, more and more studies have been available confirming the effectiveness of the therapy [22,139,140,148,149]

## Data Availability

No new data were created.
